# Juxta-articular Myxoma of the Hip: A Rare Pediatric Tumor

**DOI:** 10.5435/JAAOSGlobal-D-19-00070

**Published:** 2019-11-12

**Authors:** Alessandro Raffaele, Ilaria Goruppi, Mario Mosconi, Francesco Pelillo, Marco Lucioni, Francesco Benazzo, Luigi Avolio

**Affiliations:** From the Pediatric Surgery Unit (Dr. Raffaele, Dr. Goruppi, and Dr. Avolio), the Orthopaedic and Traumatology (Dr. Mosconi, Dr. Pelillo, and Dr. Benazzo), and the Division of Pathology (Dr. Lucioni), Fondazione IRCCS Policlinico San Matteo and University of Pavia, Pavia, Italy.

## Abstract

Juxta-articular myxoma (JAM) is a very rare myxoid tumor in pediatric age. JAM is frequently located at large joints, particularly in the knee, and most lesions present as palpable swelling sometimes associated with pain or tenderness. Only three cases of pediatric JAM have been reported in the literature to date. We describe a case of JAM in a 12-year-old boy, characterized by several unique aspects related to age, site of onset, size, and associated symptoms.

Juxta-articular myxoma (JAM) is an uncommon benign tumor occurring between third and fifth decade of life.^1^ The tumor is located most frequently at large joints (knee in 88% of cases).^2^ Only three cases of pediatric JAM have been reported in the literature to date.

We present a case of JAM of the hip in a 12-year-old boy with several unique aspects.

## Case Report

**Figure 1:**
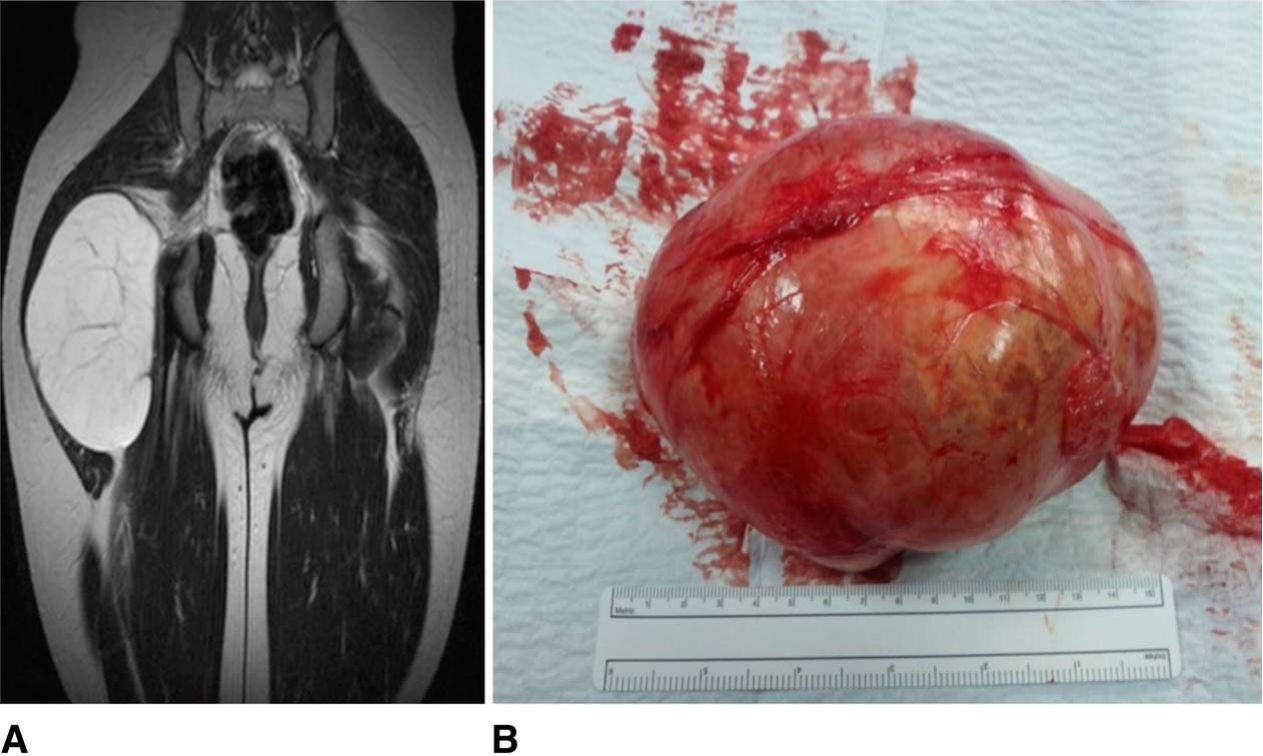
**A**, Pelvic MRI, T2 sequence, coronal. Close to the right greater trochanter, massive hyperintense lesion localized between the gluteus medius and the gluteus maximus. The lesion compresses the gluteus maximus on the right. **B**, Intraoperative appearance of the lesion.

A 12-year-old boy was admitted because of the sudden appearance and increase in size in few days of a large swelling of the right buttock without functional limitations of the hip. The child was in good health and denied any recent trauma. Physical examination showed a right buttock lesion with a tight, but not painful, flexible consistency; superficial venous prominence or discoloration of the skin was absent. The range of motion of the hip was complete for both active and passive movements and free from pain. The patient had no lameness nor any painful symptoms in performing daily activities. Biochemical investigations including complete blood count, liver and kidney function, inflammatory markers, and electrolytes were normal. No bone involvement at right hip radiograph. Ultrasonography showed, in comparison with another done before admittance, a complex mass with solid and cystic components increased in size of about 5 cm in 3 days. At lower-limb contrast MRI, evidence of an oval lesion, measuring 10 × 15 × 8 cm, located between the gluteus medius and the gluteus maximus. The lesion was well-capsulated, with branches and septa inside, weak endostructural diffusion, and wall irregularities with no infiltrative phenomena into surrounding tissues (Figure 1). Total-body scintigraphy showed no metabolic activity at the level of the lesion.

**Figure 2:**
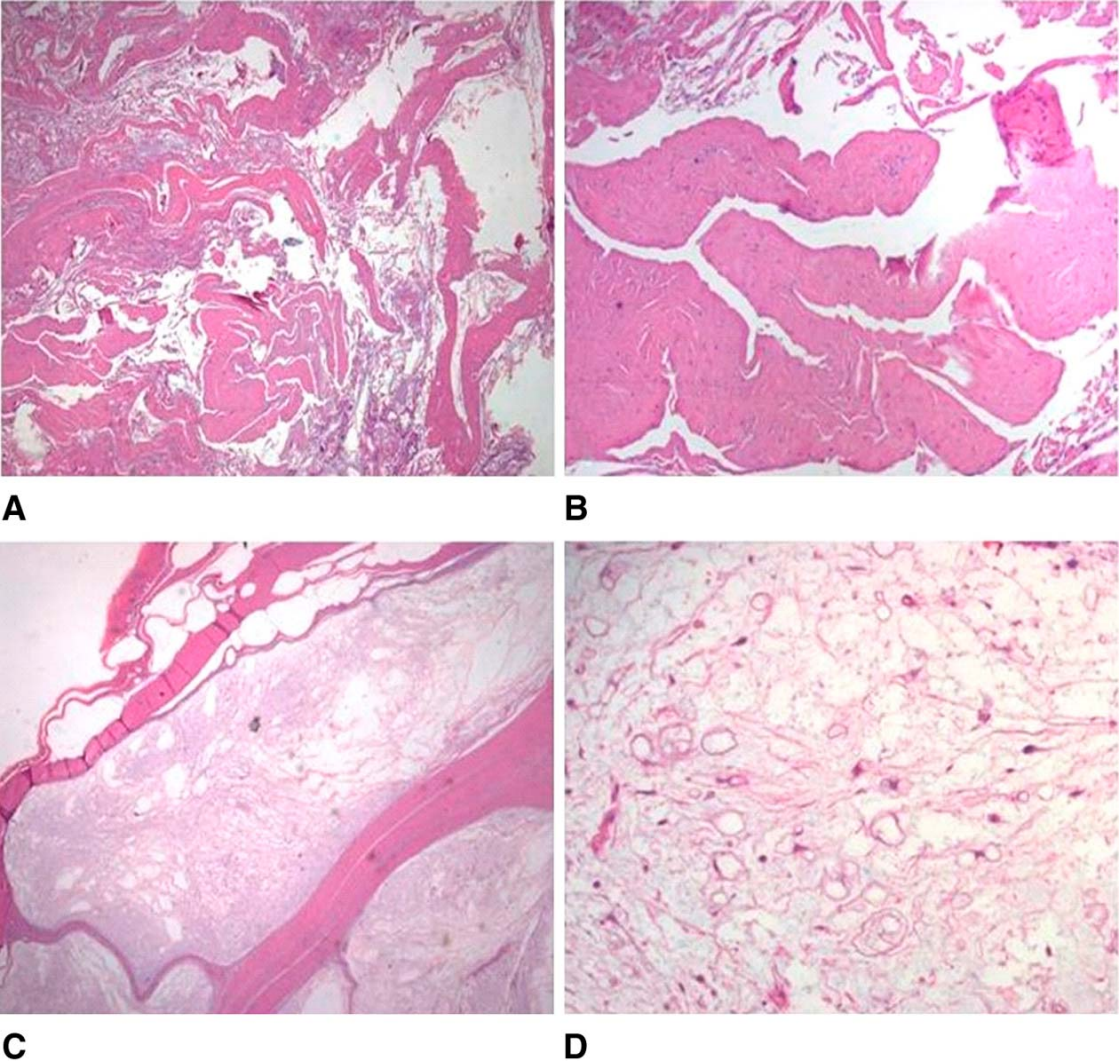
Histological appearance of the lesion with hematoxylin and eosin (HE) strain. Incisional biopsy (HE, 20×; inset 100×): strands of fibrous tissue, with scattered lymphohistocytic infiltrate (**A** and **B**). Surgical excision sample (HE, 20×; inset 200×): variously sized, thick-walled cystic spaces (**C**). At high magnification, the lesion resulted to be composed of scattered, small, oval to spindle fibroblast-like cells embedded in a richly myxoid matrix (**D**).

An open incisional biopsy was done: histopathological analysis documented strands of fibrous tissue, with scattered lymphohistocytic infiltrate, as found in a chronically inflamed articular bursa (Figure 2). In consideration of this diagnosis, the child was prescribed rest and nonsteroidal anti-inflammatory therapy. Since failure of conservative treatment (no reduction of the mass), 2 months later, we proceeded to excision of the lesion.

The resected tumor measured 10 × 17 × 8 cm, was capsulated, and showed a mucoid multinodular appearance on cut section (Figure 1). At pathological examination, the lesion consisted of variously sized, thick-walled cystic spaces; at high magnification, the lesion was composed of scattered, small, oval to spindle fibroblast-like cells embedded in a richly myxoid matrix, without cytologic atypia. The final diagnosis was JAM (Figure 2).

The postoperative course was uneventful. Five years after surgery, the patient results free from relapse.

## Discussion

JAM is an unusual benign lesion occurring between third and fifth decade of life^1,2^ belonging to myxoid tumors, lesions characterized by abundant myxoid stroma and poor cellularity and intralesional vascularization. Histologically, JAM shows an abnormal accumulation of mucinous stroma and cellularity composed almost exclusively of mesenchymal cells. Regarding management and prognosis, to distinguish JAM from other benign and malignant entities is essential.^3^ Complete surgical excision is the mainstay of treatment because local recurrence rate is estimated on 34%.^1,4^ The main differential diagnoses include intramuscular myxoma, myxoid malignant fibrous histiocytoma (myxofibrosarcoma), low-grade fibromyxoid sarcoma, myxoid liposarcoma, and ganglion cyst, of which characteristics are resumed in Table 1.

**Table 1:** Main Differential Diagnoses of Juxta-articular Myxoma

Tumor		Symptoms	Site	Histology	Age	Prognosis
Intramuscular myxoma^5^		Slowly growing, painless mass	Large muscles of the thigh, shoulder, buttock, and upper arm	Interstitial mucin, sparse spindle-shaped stromal cells, strands, or trabeculae of fibrous tissue.	Adults	Rare local recurrence, no risk for metastasis
					F > M	
Myxofibrosarcoma^6^		Painless, progressively enlarging single nodule (10 cm), growing over several months	Subcutis, not related to joints, trunk, pelvis, head and neck, and genitalia	Cellular atypia, association of myxoid areas and cellular areas in variable proportions	Adults	Aggressive, high local recurrence rate, notable metastatic rate
Low-grade fibromyxoid sarcoma^7^		Indolent clinical course	Muscle lower limb, pelvic girdle, rarely upper limb and shoulder girdle	Alternating dense fibrous tissue hypocellular myxoid nodules and more cellular areas around small blood vessels	Young adults and children	Substantial rate of local recurrence and metastasis
Myxoid liposarcoma^8^		Slow growing painless mass >10 cm at diagnosis	Lower limb, retroperitoneum (rare)	Low grade: hypocellular, bland spindle cell proliferation set in an abundant myxoid background, presence of a thin-walled, capillary-sized vascular network, organized in a distinctive plexiform pattern. High grade: hypercellular areas, exceeding 5% of the lesion.	Young adults	Low-grade metastatic risk <10%. High grade and necrosis unfavorable outcome
					M > F	
Ganglion cyst^9^		Asymptomatic groin mass, vascular compression, femoral thrombosis, and sciatica symptoms. No medical history of trauma	Joint capsules, tendon sheaths, and ligaments, hip and groin	Mixomatous degeneration of fibrous tissue structures do not have a lining of synovial cells on the cyst wall	Adults and children F > M	Mild risk of recurrence

In adults, the most frequent JAM localization is the knee (88% of cases),^2^ followed by the elbow and shoulder, rarely ankle and wrist, and less frequently hip. Causes of JAM remain controversial, although the most credible hypothesis is post-traumatic or postarthritis disease.^10^ In some cases, the pain was present even with no clinically detectable lesions, while in some others, swelling without pain was present.

JAM is a very rare tumor in children with only three pediatric cases described in the literature; in two cases, the tumor was located in the knee^2^ and one in the wrist (Table 2). JAM has never been reported as hip tumor in a pediatric patient. The presence of pain could help the differential diagnosis, even if children may have difficulty to locate the site of the pain, and sometimes, pain referred to hip could underlie knee or foot disease.

**Table 2:** Summary of Reported Cases in Children Sorted by Age, Specifying Sex, Age, Location, Size of the Myxoid Lesion, and Relation With Pain

Author		Sex	Age (y)	Localization	Diameter (cm)	Pain
Körver et al^2^		M	5	Knee	4	No
Daluiski et al^11^		F	9	Knee	3	Yes
Ozcanli et al^12^		M	16	Wrist	2	Yes

Differential diagnosis of JAM involving the hip includes pigmented villonodular synovitis, hemophilia, nonspecific synovitis, juvenile idiopathic arthritis, synovial (osteo)chondromatosis, lipoma arborescens, and synovial hemangioma.^13^ Other pathologies that could involve a hip joint are slipped femoral epiphysis, osteomyelitis, leukemia, osteoid osteoma, Ewing tumor, and osteosarcoma. All these entities may have similar clinical findings, presenting as hip pain or a limp.

Several findings of our patient are similar to the other reported pediatric cases as like as medical history negative for both inflammatory diseases and trauma. Our patient was completely asymptomatic: In one of the two previously reported pediatric cases, the tumor was found in the knee without evidence of pain, while in the other two cases, the children presented with a painful mass.^2,11,12^

A peculiar observation in our case was the dimensional discrepancy between the preoperative imaging and the excised mass, 2 cm bigger than expected. These data may indicate a rapid lesion growth, as previously described,^2,11^ or compression effect caused by surrounding tissues, which may have led us to underestimate the size based on the MRI scan.

In our case, as like as in previously reported 5-year-old boy case, biopsy was inefficacious because of collection of nonrepresentative tissue. Recently, Agrawal et al^14^ reported fine needle aspiration citology of pediatric soft-tissue lesions and neoplasms as a first-line investigation. Anyway, in his study, histopathology provided more accurate subtyping than fine needle aspiration in 8/25 cases, all benign diagnoses as myxoma and lipoblastoma. We believe that even incisional biopsy can be misleading, thereby, delaying a correct diagnosis, while to differentiate JAM from other malignant entities is mandatory, and this could guide to a more aggressive approach. Surely, this point requires a higher debate.

Wide consensus about high recurrence of myxoid tumors in case of incomplete resection is found, and postoperative follow-up is important to detect relapse. As recurrence usually occurs within 18 months,^1^ a minimum period of 2-year follow-up should be considered.

## Conclusion

Management of children with JAM requires a multidisciplinary approach and long-term postoperative follow-up. In case of hip tumefaction without pain, JAM could be considered for a diagnosis, and a malignant entity should be excluded. Adequate treatment requires surgical removal of the entire mass. Preoperative biopsies seem to be misleading for diagnosis in the pediatric age group.
